# NiS_2_@rGO Nanosheet Wrapped with PPy Aerogel: A Sandwich-Like Structured Composite for Excellent Microwave Absorption

**DOI:** 10.3390/nano9060833

**Published:** 2019-05-31

**Authors:** Zhi Zhang, Qi Lv, Yiwang Chen, Haitao Yu, Hui Liu, Guangzhen Cui, Xiaodong Sun, Ling Li

**Affiliations:** Key Laboratory of Science and Technology on Electromagnetic Environmental Effects and Electro-optical Engineering, The Army Engineering University, Nanjing 210007, China; zhangnjjn@163.com (Z.Z.); lq20190410@126.com (Q.L.); chenyw1357@163.com (Y.C.); yu1245775230@163.com (H.Y.); liuhh1005@163.com (H.L.); cgzovezy@163.com (G.C.)

**Keywords:** graphene, polypyrrole, sandwich-like structure, electromagnetic absorption

## Abstract

To reduce electromagnetic pollution as well as increase the accuracy of high-precision electronic equipment, more attention has been paid to new electromagnetic wave (EMW) absorbing materials, which have the advantages of strong absorption, wide absorption bands, and a narrow thickness. In this study, a novel ternary type of the NiS_2_@rGO/polypyrrole (PPy) sandwich-like structured composites was synthesized via a facile two-step method, in which the hydrothermal method was used to prepare NiS_2_@rGO binary composites and then the in situ polymerization method was used to synthesize the PPy, which acted as the outer layer of the sandwich-like structure. The morphologies and electromagnetic absorption performance of the NiS_2_@rGO/PPy were measured and investigated. A sample with 6 wt% NiS_2_@rGO/PPy loading paraffin-composite obtained an outstanding reflection loss (RL) of −58.7 dB at 16.44 GHz under a thickness of 2.03 mm. Simultaneously, the effective electromagnetic wave absorption bandwidth for RL < −10 dB, which covered 7.04 to 18.00 GHz (10.96 GHz), was achieved by changing the thickness of the absorber from 2.0 to 3.5 mm. The results not only suggest that the NiS_2_@rGO/PPy composite has excellent performance in the field of EMW absorption but also prove that the novel sandwich-like structure can contribute to appropriate impedance matching through multiple relaxation and interfacial polarization processes.

## 1. Introduction

In recent years, with the development of advanced high-power electronic equipment, electromagnetic (EM) pollution, which has harmful effects on human life, has become significant [1,2,3]. In this regard, more attention has been focused on developing highly efficient and stable electromagnetic wave (EMW) absorbing materials to solve EM pollution [4]. The practical application of single-component absorbers, such as Fe_3_O_4_, Co, and polypyrrole (PPy), is restricted because they do not have the characteristics of small matching thickness, light weight, strong absorption features, and a wide EM absorption band. In contrast with single absorbers, multiple-component absorbers can better meet the requirements of being a highly efficient EMW absorber on account of their adjustable impedance matching and multilayer structures [5,6].

Reduced graphene oxide (rGO), a new type of two-dimensional material, has shown a promising application prospect in the field of EMW absorption [7,8]. On the one hand, a large specific surface area will increase the transmission intensity of electromagnetic waves in rGO-based composites and help EM waves get inside the rGO-based composites. EM waves will be consumed significantly by the synergistic effects of interlaminar multiple refraction, reflection and surface fold scattering [9,10]. On the other hand, the surface area and polar functional groups distributed on rGO undergo polarization relaxation under an external electric field, giving it a strong dielectric loss [11,12,13]. For example, Fang et al. studied three-dimensional rGO powder and found that the 3D-rGO shows efficient microwave absorption in the S-band due to its honeycomb-like structure and the strong polarization [14]. However, a single rGO EMW absorber cannot achieve good impedance matching due to its simple dielectric loss mechanism. Some reports have proved that combining a low dielectric component with rGO can balance the dielectric constant of the composite and obtain appropriate impedance matching [15,16]. Davide Micheli et al. studied carbon-based nanostructures and their nanocomposites with different sizes and morphologies, at the same time, they evaluated the electromagnetic absorption properties of carbon-based nanocomposites [17,18]. Their work also proved that rGO-based composites with a graded-dielectric multilayer has an excellent EM absorption potential [19]. Wang et al. prepared MnO_2_-rGO nanocomposites via a hydrothermal method and the heterostructure exhibited the highest reflection loss (RL) of −37 dB at 16.8 GHz [20]. The research group of Zhao and Ji used an in situ synthesis method to fabricate an ACNT/rGO/BaFe_12_O_19_ composite. The minimum reflection loss value of the ACNT/rGO/BF composite was −19.03 dB at 11.04 GHz with a frequency bandwidth of 3.8 GHz [21]. The narrow effective frequency bandwidth and large thickness will limit the further application of rGO-based binary composites. To further solve this problem, it is feasible to embellish rGO-based binary composites on the conducting polymer.

Conducting polymers such as PPy have attracted the attention of many research laboratories due to their unusual properties, including low density, stable chemical properties, and high conductivity [22,23,24]. Recently, extensive studies have proved that polypyrrole can be used as an effective material in the EMW absorption field [25,26]. This is due to the synergistic effects of the conjugated bonds and electric dipoles in the composites containing PPy under external EMW, which could improve the dielectric loss and impedance matching. For example, Chen et al. synthesized a novel ternary graphite/polyaniline/CoFe_2_O_4_ nanocomposites and found that the maximum reflection loss was around −19.13 dB at 13.28 GHz with a thickness of 0.5 mm [27]. Liu et al. synthesized uniform core-shell PPy@C composites with a highest reflection loss of −38.1 dB at 11.6 GHz [28]. Their work proved that adding an additional interfacial polarization step can play a role in improving impedance matching if the carbon-based nanomaterials are coated with PPy. Inspired by this, the rGO-based binary composite coated with PPy has great potential to obtain new types of EMW absorber with a wide absorbing frequency, light weight, and strong absorption.

In the current study, we fabricated a novel ternary type of the NiS_2_@rGO/polypyrrole (NiS_2_@rGO/PPy) sandwich-like structure composite with a remarkable EMW absorption property. A NiS_2_@rGO binary composite was synthesized by a facile hydrothermal method, to act as the inner layer of the sandwich-like structure. The outside layer of the sandwich-like structure was formed by the PPy which was produced by the in situ polymerization method [29]. In this work, NiS_2_ supported by rGO formed the electric polarization center of the composite, giving it a controllable particle size and low dielectric constant and adjusting impedance matching by producing an interface polarization with rGO. At the same time, PPy acted as the shell of the sandwich-like structure, synergizing with NiS_2_@rGO and consuming EMW by scattering and multiple refraction in sandwich-like structures. The detailed EM absorption performance study on the NiS_2_@rGO/PPy sandwich-like structure composite showed an excellent intensity and bandwidth for EM absorption as well as good compatibility and excellent stability during production and testing.

## 2. Experimental Section

### 2.1. Materials

Nickel chloride hexahydrate (NiCl_2_·6H_2_O), polyvinyl pyrrolidone (PVP-30,000), sulfur powder, and pyrrole (Py) were obtained from Aladdin Industrial Corporation (Shanghai, China). GO powder was provided by Xianfeng Chemical Co. Ltd. (XF002-2, Nanjing, China). All of the reagents were of analytical grade and were used without further purification.

### 2.2. Synthesis of NiS_2_@rGO Binary Composite

The NiS_2_@rGO binary composite was prepared through a facile hydrothermal method [30,31]. Firstly, 0.05 g of GO powder was added into 50 mL of deionized water and then sonicated for 60 min at 20 °C (120 W, 40 KHz). After that, the GO dispersed solution continued to be magnetically stirred at 70 °C for further processing. Secondly, 0.9506 g of NiCl_2_·6H_2_O dissolved in 30 mL of deionized water was added to the GO dispersed solution. A quantity of 0.213 g of sulfur powder was dissolved in 30 mL of deionized water under sonication for 10 min at 20 °C to form a S dispersed solution and then added into the GO mixture solution. Then, 1.6 g polyvinyl pyrrolidone (PVP-30,000) was dissolved in 10 mL deionized water and slowly added into the abovementioned GO mixture solution. After that the mixture solution magnetically stirred for 20 min at 20 °C to mix well. Subsequently, the mixture was placed in a 160 mL Teflon-lined stainless-steel autoclave and maintained at 200 °C for 12 h. After this reaction, the obtained product was washed with deionized water and ethanol several times and vacuum dried at 60 °C for 14 h.

### 2.3. Synthesis of NiS_2_@rGO/PPy Sandwich-Like Structure Composite

The NiS_2_@rGO/PPy sandwich-like structure composites were synthesized as described in the suit method [32]. Briefly, 40 mg of the NiS_2_@rGO binary composite synthesized in the previous step was dissolved in a mixture of 4 mL deionized water and 4 mL ethanol to form a NiS_2_@rGO dispersed solution and sonicated for 10 min. Then, 135 mg of pyrrole was slowly trickled into the aforementioned solution. After that, 2 mL of deionized water and 2 mL of absolute ethanol was used to fully dissolve 1.6 g FeCl_3_·6H_2_O, and the FeCl_3_·6H_2_O solution was stepwise injected into the NiS_2_@rGO/Py solution under swift mechanical stirring for 5 min. A fluffy black powder was obtained after the NiS_2_@rGO/Py solution had reacted for 24 h. Finally, the black NiS_2_@rGO/PPy composites were rinsed with absolute ethanol and distilled water several times until the filtrate was colorless, and then, they were dried in a vacuum at 50 °C for 24 h. In addition, the NiS_2_@rGO/PPy with 40, 60, and 80 mg of NiS_2_@rGO were used for comparative analysis, which are recorded as samples A, B and C, respectively.

### 2.4. Characterization and Electromagnetic (EM) Parameters Measurement

X-ray diffraction (XRD) was performed using a Bruker D8 Advanced X-ray diffractometer (BRUKER, Karlsruhe, Germany) with Cu K_α_ radiation (λ = 1.5406 Å) at 40 KV over the range of 2θ = 5–80°. The Fourier transform infrared (FT-IR) spectra of the samples were characterized by a Nicolet IS10 FT-IR spectrometer with KBr and the field emission scanning electron microscopy (FE-SEM) data of the samples were analyzed using a Hitachi S4800 field emission scanning microscope. The further morphology, crystal structure, and element distribution properties of the NiS_2_@rGO/PPy composite were determined by transmission electron microscopy (TEM) with an energy-dispersive X-ray spectroscope (EDS) and a high-resolution transmission electron microscope (HRTEM), which were provided by JEL-2100F (JEOL, Tokyo, Japan) and Tecnai G^2^ F20 (FEI Company, Hillsboro, OR, USA), respectively. X-ray photon spectroscopy (XPS) was performed on an ESCALAB 250 (Thermo, Waltham, MA, USA).

### 2.5. EM Absorption Measurement

The microwave scattering parameters of the samples composing of paraffin wax NiS_2_@rGO/PPy composite were studied using an Agilent N5224A PNA (Agilent Technologies Inc, Santa Clara, CA, USA) vector network analyzer (VNA) recorded at 2–18 GHz. Samples composed of wax and NiS_2_@rGO/PPy composite were fabricated into cylindrical compacts (Φ_out_ = 7.00 mm, Φ_in_ = 3.04 mm) with different proportions of NiS_2_@rGO/PPy (4, 6, and 8 wt%). The VNA operates based on the transmission line theory, and was used to calculate the electromagnetic parameters of the sample by measuring four scattering parameters (S_11_, S_22_, S_12_, and S_21_) of the two-port network [33,34,35]. The electromagnetic parameter testing principle is that when an electromagnetic wave is incident on an absorbing material with a certain thickness, it will generate multiple reflections and transmits. The schematic diagram of the fixture and the test sample is illustrated in Figure S1.

## 3. Results and Discussion

**Scheme 1:**
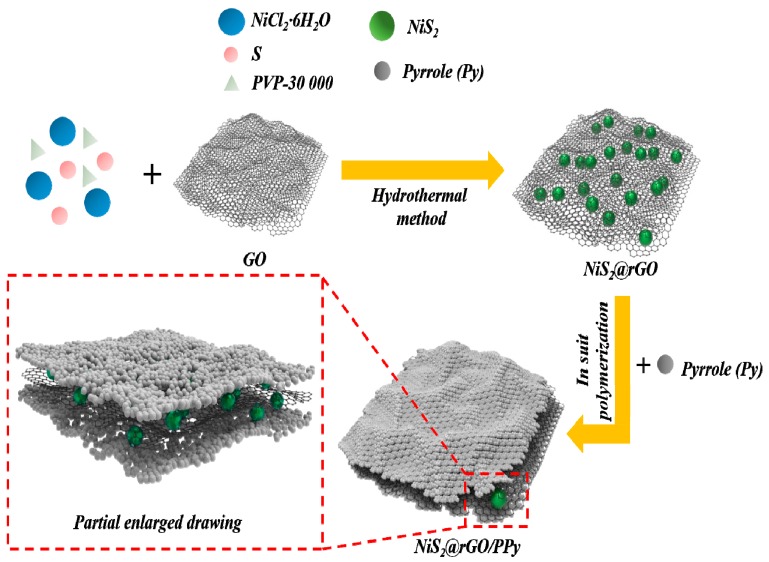
The preparation process of the NiS_2_@rGO/polypyrrole (PPy) sandwich-like structure composite.

The NiS_2_@rGO/polypyrrole (NiS_2_@rGO/PPy) sandwich-like structure composite was synthesized by a facile two-step method. Fitstly, the NiS_2_@rGO binary composite was prepared by a hydrothermal method. Secondly, an in suit method was used to synthesize the NiS_2_@rGO/PPy sandwich-like structure composites on the basis of NiS_2_@rGO binary composite. The synthetic procedure of the NiS_2_@rGO/PPy sandwich-like structure composite is illustrated in Scheme 1.

### 3.1. Characterization of Samples

**Figure 1:**
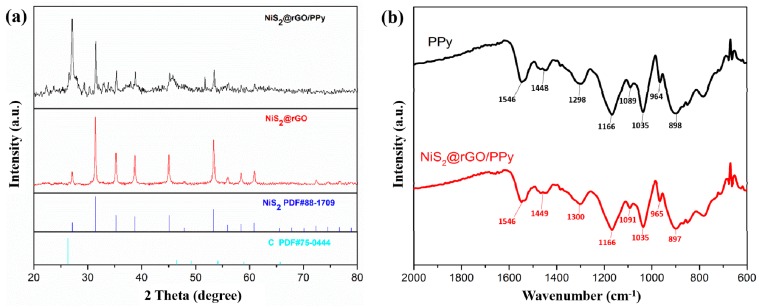
(**a**) X-ray diffraction (XRD) patterns of the NiS_2_@rGO/PPy ternary composite and the NiS_2_@rGO binary composite; (**b**) Fourier transform infrared (FT-IR) spectra of the PPy and NiS_2_@rGO/PPy ternary composites.

In order to investigate the crystal structure of the NiS_2_@rGO/PPy ternary composite and the NiS_2_@rGO binary composite, the XRD patterns are presented in Figure 1a. The existence of NiS_2_ in the NiS_2_@rGO composite can be clearly confirmed, because all of the diffraction peaks correspond with the standard card of NiS_2_ (#88-1709). Furthermore, the XRD pattern of NiS_2_@rGO/PPy ternary composite is almost consistent with the standard card of NiS_2_ (#88-1709) and the standard C card (#75-0444), while the characteristic diffraction peaks are broad and weak due to the existence of amorphous PPy, which is indicative of poor crystallization. The XRD pattern of GO is presented in Figure S2. To verify the existence of PPy, the surface chemical states of the NiS_2_@rGO/PPy ternary composite were further investigated via FT-IR. The FT-IR spectra of PPy and NiS_2_@rGO/PPy are shown in Figure 1b. Figure 1b shows that for NiS_2_@rGO/PPy, the characteristic bands at 1546 and 1449 cm^−1^ are associated with symmetrical and anti-symmetrical ring-stretching modes, respectively, whereas the peaks located at 1300 and 1035 cm^−1^ correspond to C–N stretching vibrations and the C–H band in-plane vibration of PPy rings, respectively [36]. At the same time, the doping state of PPy can be verified by the characteristic peaks at 1166 and 897 cm^−1^, and the existence of PPy can be proved by the peaks located at 1091 and 965 cm^−1^ [37]. These characteristic peaks in the FT-IR spectra of NiS_2_@rGO/PPy correspond well with the FT-IR spectra of PPy, which could be associated with the successful coating of PPy.

**Figure 2:**
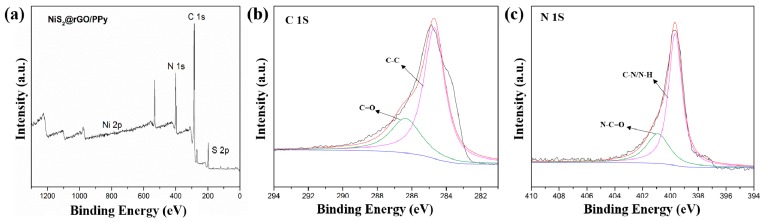
X-ray photon spectroscopy (XPS) spectra of NiS_2_@rGO/PPy ternary composite (**a**); XPS core-level spectra of C 1s (**b**) and N 1s (**c**) of NiS_2_@rGO/PPy.

The chemical composition of the NiS_2_@rGO/PPy ternary composite was also determined by XPS (Figure 2). The peaks of four elements (Ni, N, C, and S) are clearly displayed in Figure 2a, which further confirms that NiS_2_@rGO/PPy ternary composite was successfully synthesized. In addition, although the characteristic peaks of N and C are obvious, the characteristic peaks of Ni 2p and S 2p are weak because the NiS_2_ nanospheres coated with rGO and PPy were placed in the innermost layer of the composite structure, which is difficult to characterize and analyze accurately using XPS. As shown in Figure 2b, in the spectrum of C 1 s, the major peaks at 284.5 and 286.4 eV are attributed to the C–C bonds of the aromatic rings and the C=O bonds of the carbonyl, respectively. The intensity of the C=O bonds is subdued, which could indicate that the GO was reduced [38]. N 1s spectra are exhibited in Figure 2c, in which the peaks located at 399.65 and 400.9 eV are assigned to the C–N/N–H bonds and the N–C=O bonds, respectively [39,40]. The chemical composition of GO was determined by XPS (Figure S3a), and the core-level spectrum of C 1S in GO is displayed in Figure S3b. The phase analysis combining XRD, FT-IR and XPS spectra accurately proves that NiS_2_, rGO, and PPy successfully formed a new type of ternary composite without doping with any other substances.

**Figure 3:**
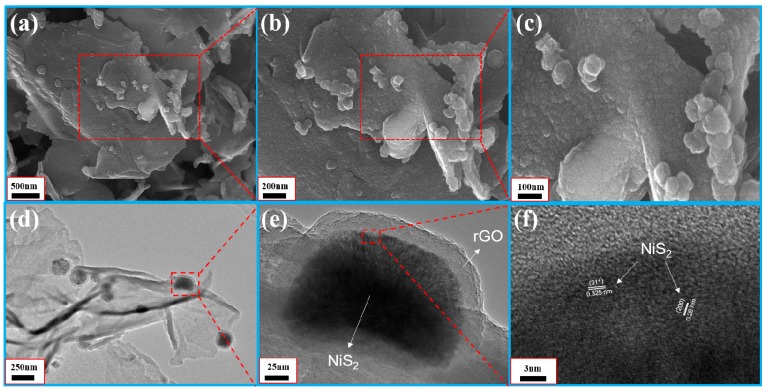
Scanning electron microscope (SEM) images of the NiS_2_@rGO/PPy ternary composite (**a**–**c**); transmission electron microscope (TEM) images of NiS_2_@rGO/PPy ternary composite (**d**,**e**); high-resolution TEM (HRTEM) images of the NiS_2_@rGO/PPy ternary composite (**f**).

**Figure 4:**
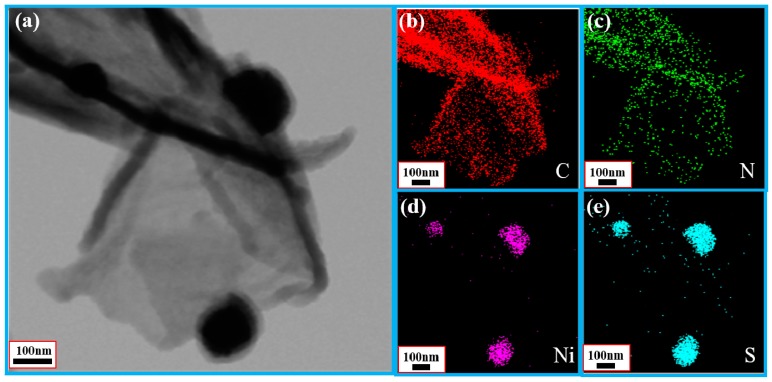
TEM image (**a**) and energy-dispersive X-ray spectroscopy (EDS) mapping images (**b**–**e**) of the NiS_2_@rGO/PPy ternary composite.

In this study, to investigate the microstructures and morphologies of the composites, typical SEM and TEM images of the NiS_2_@rGO/PPy ternary composite were made and are presented in Figure 3. Figure 3a–c show SEM images of the NiS_2_@rGO/PPy ternary composite at different resolutions, and the EDS pattern has been presented in Figure S4b. It can be clearly observed that the PPy nanospheres uniformly coated the NiS_2_@rGO binary composites while maintaining the integrity of the binary composites, forming a complete sandwich-like composite structure. Meanwhile, at the edge of the sandwich-like structure, there is a slight aggregation of PPy nanospheres. As revealed by Figure 3b,c, the average thickness of the sandwich-like NiS_2_@rGO/PPy ternary composite is about 100 nm, which is mainly comprised of PPy nanospheres. The SEM images of NiS_2_@rGO is presented in Figure S5. The TEM pattern presented in Figure 3d accurately shows the microstructure of the inner layer of the sandwich-like NiS_2_@rGO/PPy composite, in which the NiS_2_ nanospheres are well encased in rGO nanosheets. The diameter of the inner NiS_2_ nanospheres is approximately 200 nm while the thickness of the out-layer rGO nanosheets is about 20 nm (Figure 3e). Such unique structures allow the NiS_2_ nanospheres to fully interact with rGO nanosheets to significantly adjust the dielectric properties of the whole composite. In order to further validate the existence of NiS_2_ nanospheres, the HRTEM pattern of NiS_2_@rGO/PPy composite is displayed in Figure 3f. The lattice planes were calculated to be 0.325 and 0.28 nm, which match well with the (200) and (311) planes of NiS_2_, respectively. The distribution of elements in the sandwich-like NiS_2_@rGO/PPy ternary composite is shown by the EDS spectra in Figure 4, from which C, N, Ni, and S were measured. The distributions of the measured Ni and S elements validate the hypothesis that the NiS_2_ nanospheres are encased in rGO nanosheets; meanwhile, the distributions of the detected C and N elements further verify the unique sandwich-like structure. The morphology analysis, which combined SEM, TEM and EDS spectra, further validated that the new ternary composite composed with NiS_2_, rGO and PPy was synthesized with a unique sandwich-like structure to obtain an excellent EMW performance.

### 3.2. Electromagnetic Parameters of the NiS_2_@rGO/PPy Composite

**Figure 5:**
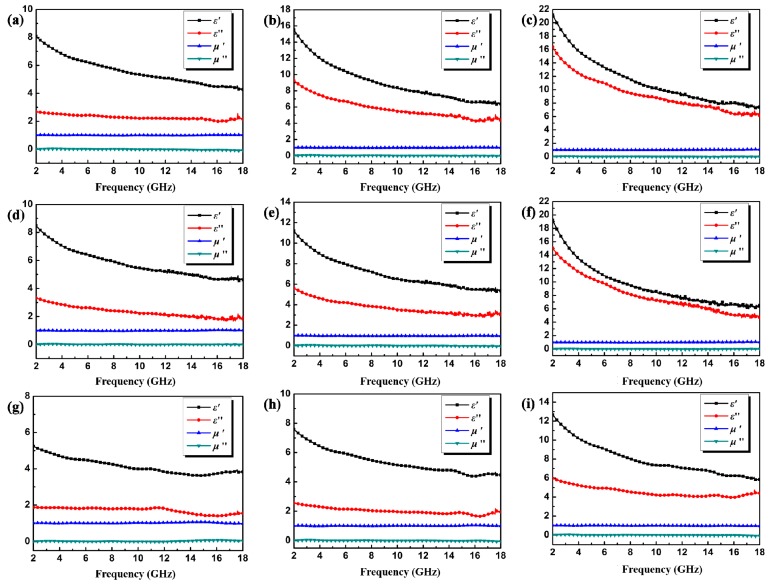
Frequency dependence of the ε′, ε″, μ′, and μ″ values of sample A with filler loadings of 4 (**a**), 6 (**b**), and 8 wt% (**c**); sample B with filler loadings of 4 (**d**), 6 (**e**), and 8 wt% (**f**); and sample C with filler loadings of 4 (**g**), 6 (**h**), and 8 wt% (**i**).

To investigate the EMW absorption properties of the NiS_2_@rGO/PPy composite, real parts of complex permittivity (ε′), imaginary parts of complex permittivity (ε″), real parts of complex permeability (μ′), and imaginary parts of complex permeability (μ″) of samples A, B, and C with filler loadings of 4, 6, and 8 wt% were measured at room temperature. The real parts (ε′ and μ′) and imaginary parts (ε″ and μ″) of complex permittivity and permeability represent the storage ability of microwave energy and the dissipation ability of electric and magnetic energy, respectively [15]. Figure 5 shows that the value of μ′ and μ″ are appropriately constant and close to 1 and 0, respectively. There are no significant changes in the numerical values of the frequency-dependent complex permeability values (μ_r_) of the samples calculated by the real and imaginary parts of complex permeability (μ_r_ = μ′ − jμ″), which is associated with the non-magnetic characteristics of the as-prepared materials. On the contrary, for the dielectric properties of the as-prepared materials, the frequency dependent complex permittivity values (ε_r_, ε_r_ = ε′ − jε″) of the NiS_2_@rGO/PPy composite in paraffin matrix was measured at 2–18 GHz, as shown in Figure 5. In fact, numerous reports have indicated that in order to obtain a fine impedance match, a relatively moderate dielectric constant is needed, and the relatively higher or lower permittivity will cause the EMW to be reflected out of the absorber [16]. With the increase in the content of rGO-based binary composites, the high permittivity of PPy was effectively regulated, which is in favor of EMW absorption of NiS_2_@rGO/PPy. According to the free-electron theory, the relationship between the electrical resistivity (ρ) and ε″, and the relationship between electrical conductivity (σ) and skin depth (δ) can be expressed using the following equation [15]:

(1) $$\varepsilon^{\prime \prime }\approx 1/\pi \varepsilon_{0}\rho f$$

(2) $$\delta =1/\sqrt{\pi f\mu \sigma }$$

where ƒ is the frequency of EMW, ε_0_ is the dielectric constant in vacuum (ε_0_ = 8.854 × 10^−12^ F m^−1^). It can be found that lower ε″ bring about higher resistivity of NiS_2_@rGO/PPy composite, and lower conductivity could signifies a big skin depth, eventually increase transmittance and reduce reflection of the EM wave. Thus, a higher electrical resistivity will obtain a better electromagnetic absorption performance; and a relatively lower ε″ might be required. With the increase of filler loading, the value of ε′ and ε″ of the three samples also show an increasing trend. By contrast, with the increase of NiS_2_@rGO content, the value of ε′ and ε″ show an downward trend in every filler loading (4, 6, and 8 wt%). As shown in Figure 5, the ε″ values of all samples decrease with an increase in frequency from 2 to 18 GHz, which indicates that the NiS_2_@rGO/PPy composite might have an excellent EMW-absorbing effect at high frequencies.

**Figure 6:**
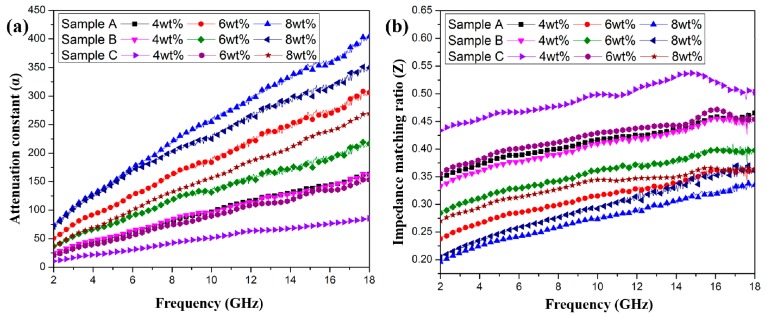
Frequency dependence of the attenuation constant (**a**) and impedance matching ratio (**b**) of samples A, B, and C with different filler loading ratios.

To obtain new types of EMW absorptive materials with strong absorption and wide absorption bands, not only should we think about the complex permittivity (ε_r_) and the complex permeability (μ_r_) of the as-prepared composites, but we also need to calculate the attenuation constant (*α*) and the impedance matching ratio (*Z*) [41]. In terms of the transmission line theory, the relationship between *α* and the corresponding matching frequency can be expressed through the following equation [42]:

(3) $$\alpha =\frac{\sqrt{2}\pi f}{c}\sqrt{(\mu^{\prime \prime }-\varepsilon^{\prime \prime })+\sqrt{{\left(\mu^{\prime \prime }\varepsilon^{\prime \prime }-\mu^{\prime }\varepsilon^{\prime }\right)}^{2}+{\left(\mu^{\prime }\varepsilon^{\prime \prime }+\mu^{\prime \prime }\varepsilon^{\prime }\right)}^{2}}}$$

where ƒ is the frequency of EMW, and *c* is the velocity of light in a vacuum. Meanwhile, the impedance matching ratio (*Z*) can be described by the following equations [16]:

(4) $$Z_{1}=Z_{0}\sqrt{\mu_{r}/\varepsilon_{r}}$$

(5) $$Z=Z_{1}/Z_{0}$$

where *Z_0_* ≈ 378 Ω is the free space of impedance matching, *Z_1_* is the impedance value of the absorber, *ε_r_* and *μ_r_* are the complex permittivity and permeability, respectively. On the one hand, it is obvious in Figure 6a that the overall trend of *α* decreased as the content of NiS_2_@rGO composites increased (α_A_ > α_B_ > α_C_). On the other hand, Figure 6b shows that the integral tendency of *Z* increased as the content of NiS_2_@rGO composites increased (Z_C_ > Z_B_ > Z_A_). Although Sample A possesses the best attenuation property and Sample C possesses the optimal impedance matching characteristic, the worst *Z* performance was shown by Sample A, and the poorest value of *α* was exhibited by Sample C. It is worth noting that an excellent EMW absorber should consider both impedance matching and energy conservation at the same time. For the Sample B, the value of the impedance matching ratio and attenuation constant are both in the middle of the acceptable level; thus, we predict that sample B will exhibit the most significant EMW absorption performance.

**Figure 7:**
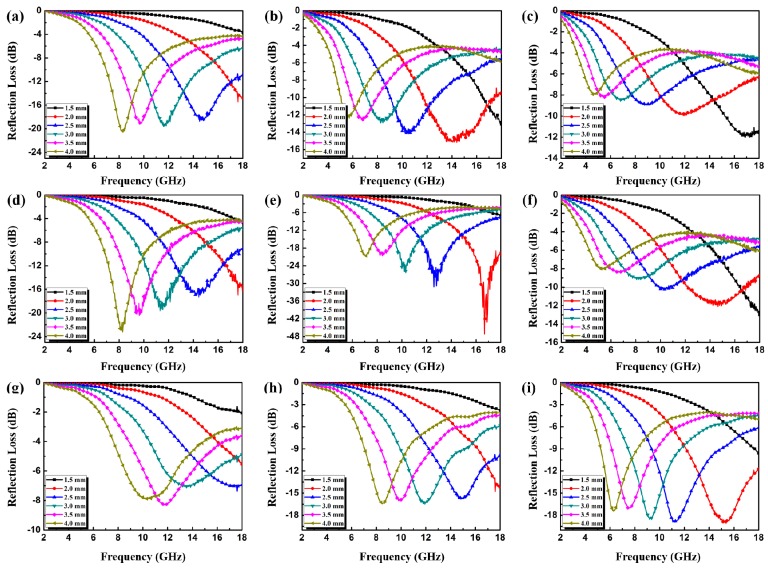
Reflection loss curves of sample A with filler loadings of 4 (**a**), 6 (**b**), and 8 wt% (**c**); sample B with filler loadings of 4 (**d**), 6 (**e**), and 8 wt% (**f**); and sample C with filler loadings of 4 (**g**), 6 (**h**), and 8 wt% (**i**).

To investigate the EMW absorption properties of the NiS_2_@rGO/PPy composite with different doping ratios, the measured complex permittivity and permeability were used to calculate the reflection loss values of the as-prepared composites by the following equation [43]:

(6) $$Z_{in}=Z_{0}\tanh\sqrt{\frac{\mu_{r}}{\varepsilon_{r}}}\left[\sqrt{\mu_{r}\varepsilon_{r}}\left(\frac{2\pi fd}{c}\right)j\right]$$

(7) $$\mathrm{RL}=20\log\left|\frac{Z_{in}-Z_{0}}{Z_{in}+Z_{0}}\right|$$

where *Z_in_* is the input characteristic impedance, *Z_0_* ≈ 378 Ω is the free space of impedance matching, ƒ is the frequency of EMW, *c* is the velocity of light in a vacuum, *d* is the thickness of the composites, and *ε_r_* and *μ_r_* are the complex permittivity and permeability, respectively. When the reflection loss value is lower than −10, −20 and −30 dB, more than 90%, 99%, and 99.9% of the EM energy is absorbed, respectively [44]. It is probable that bandwidths lower than −10 dB are effective EMW absorption bandwidths. Figure 7 shows a plot of the RL versus the frequency of the samples with different filler loading ratios at various thicknesses. For sample A (Figure 7a–c), it can be seen that the minimum reflection loss was −20.56 dB at 8.28 GHz with a thickness of 4.0 mm, and a bandwidth of RL below −10 dB is about 3.12 GHz (7–10.12 GHz), for which the thickness was too thick and the effective EMW absorption bandwidth was narrow. As shown in Figure 7g, h, and i, the lowest reflection loss value of Sample C was −19.05 dB at 15.36 GHz with a thickness of 2.0 mm, and an bandwidth of RL below −10 dB is about 5.48 GHz (12.52–18.00 GHz). Compared with sample A, the effective EMW absorption thickness was much thinner and the effective EMW absorption bandwidth was greatly improved, but the maximum EMW absorption intensity was not satisfactory. This is because of the weak attenuation capability caused by the relatively low dielectric constant. Nevertheless, it must be mentioned that a satisfactory result among the three NiS_2_@rGO/PPy composites was shown not only for the characteristics of the maximum EMW absorption intensity and the corresponding thickness but also for the effective EMW absorption bandwidth of sample B. When the filler loading ratio reached 6 wt%, the lowest reflection loss was −47.15 dB at 16.15 GHz with a thickness of 2 mm, and the broadest effective bandwidth, from 10.44 to 16.48 GHz (6.04 GHz), was obtained when the thickness was 2.5 mm. The effective EMW absorption bandwidth with a reflection loss of below −10 dB was 10.96 GHz (7.04–18.00 GHz) with a narrow thickness of 2.00–3.50 mm. In this work, the NiS_2_@rGO/PPy sandwich-like structure composites exhibited outstanding EMW absorption performance, which can be ascribed to the following reasons: firstly, the intensified polarization of the NiS_2_@rGO/PPy interface is conducive to EMW absorption; secondly, the sandwich-like structure formed by the PPy coating can increase the multiple losses of the incident EMW and effectively improve the impedance matching of the NiS_2_@rGO/PPy composites.

**Figure 8:**
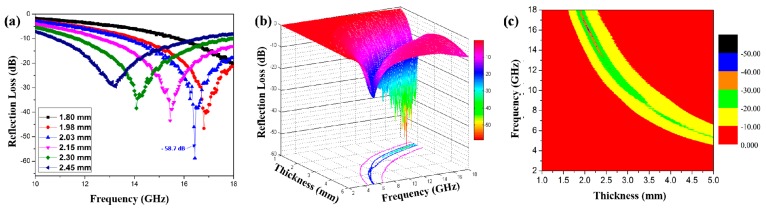
Simulation of reflection loss (RL) curves (**a**), 3D representations of RL (**b**) and the corresponding contour map (**c**) of sample B with a filler loading ratio of 6 wt% in wax composites.

Regarding the thickness of the composites, many previous studies have mentioned am important phenomenon whereby the thickness of the composites is an important index of EMW absorption because it significantly affects the RL value and the maximum absorption frequency [15,41]. Hence, to further evaluate the optimal EMW absorption properties of the NiS_2_@rGO/PPy composite, the RL curves of sample B with a thickness from 1.80 to 2.45 mm at a filler loading ratio of 6 wt% are shown in Figure 8a. Meanwhile, 3D representations of RL and the corresponding contour maps are presented in Figure 8b and c to illustrate the relationship between EM absorption frequency and the reflection loss intensity with thicknesses of 1–5 mm. As Figure 8 displays, when the thickness of sample B with a filler loading of 6 wt% was between 1.95 and 2.3 mm, all of the lowest RL values were lower than −30 dB and the corresponding maximum absorption frequency appeared in the Ku frequency range. The minimum reflection loss was –58.7 dB at 16.44 GHz with a thickness of 2.03 mm, and the bandwidth of RL below –10 dB is about 4.32 GHz (13.68–18.00 GHz) with a thickness of only 2.03 mm. It is obvious that the NiS_2_@rGO/PPy composite has an excellent performance in the field of EMW absorption. This is because the effective EMW absorption bandwidth of the as-prepared composite almost covers the entire Ku frequency range, and the characteristics of strong absorption and thin thickness make it an advanced EMW-absorbing material.

## 4. Conclusions

To sum up, a novel ternary type of the NiS_2_@rGO/PPy sandwich-like structure composites was synthesized by using a facile two-step method. The influence of the proportions of components on the EMW absorbing performance was investigated. The as-prepared compound with 60 mg NiS_2_@rGO binary composite added exhibited some distinctly strong EMW attenuation properties. From a practical point of view, this novel NiS_2_@rGO/PPy sandwich-like structure composite showed an effective EMW absorption bandwidth (RL < −10 dB) of about 10.96 GHz (7.04–18.00 GHz) with a thickness of 2.00–3.50 mm. The optimal reflection loss was −58.7 dB at 16.44 GHz for a sample with 6 wt% NiS_2_@rGO/PPy at a thickness of 2.03 mm. The superior performance of the as-prepared composites in the field of EMW absorption could be attributed to the novel sandwich-like structure and multiple relaxation and interfacial polarization processes, which contribute to the optimal compatibility of impedance matching. Therefore, it can be reasonably pointed out that the NiS_2_@rGO/PPy composites obtained in this work have the advantages of strong absorption capacity, absorption bandwidth, and narrow thickness, and has a strong application significance in preventing electromagnetic wave pollution.

## Supplementary Materials

The following are available online at https://www.mdpi.com/2079-4991/9/6/833/s1: Figure S1. Schematic diagram of the fixture (a) and the test sample (b), Figure S2. The XRD pattern of GO, Figure S3. XPS spectra of GO (a); XPS core-level spectra of C 1s in GO (b), Figure S4. SEM image of NiS_2_@rGO/PPy (a) and the corresponding EDS spectra (b), Figure S5. SEM images of NiS_2_@rGO.
