# Supplementary material for: NiS2@rGO Nanosheet Wrapped with PPy Aerogel: A Sandwich-Like Structured Composite for Excellent Microwave Absorption
Source: Nanomaterials (Basel). 2019 May 31;9(6):833. doi: 10.3390/nano9060833 (PMC6630302; doi:10.3390/nano9060833)
Supplement: Supplementary file 1 [file nanomaterials-09-00833-s001.zip › nanomaterials-502883-supplementary-for final.pdf]

Supplementary Materials:

NiS_2@rGO Nanosheet Wrapped with PPy Aerogel: A Sandwich-Like Structured Composite for Excellent Microwave Absorption

Zhi Zhang, Qi Lv, Yiwang Chen, Haitao Yu, Hui Liu, Guangzhen Cui, Xiaodong Sun* and Ling Li *

Key Laboratory of Science and Technology on Electromagnetic Environmental Effects and Electro-optical Engineering, The Army Engineering University, Nanjing 210007, China; zhangnjjn@163.com (Z.Z.); lq20190410@126.com (Q.L.); chenyw1357@163.com (Y.C.); yu1245775230@163.com (H.Y.);
liuhh1005@163.com (H.L.); cgzovezy@163.com (G.C.)

* Correspondence: xiaodongsunlgdx@126.com (X.S.); leonleeust@163.com (L.L.)

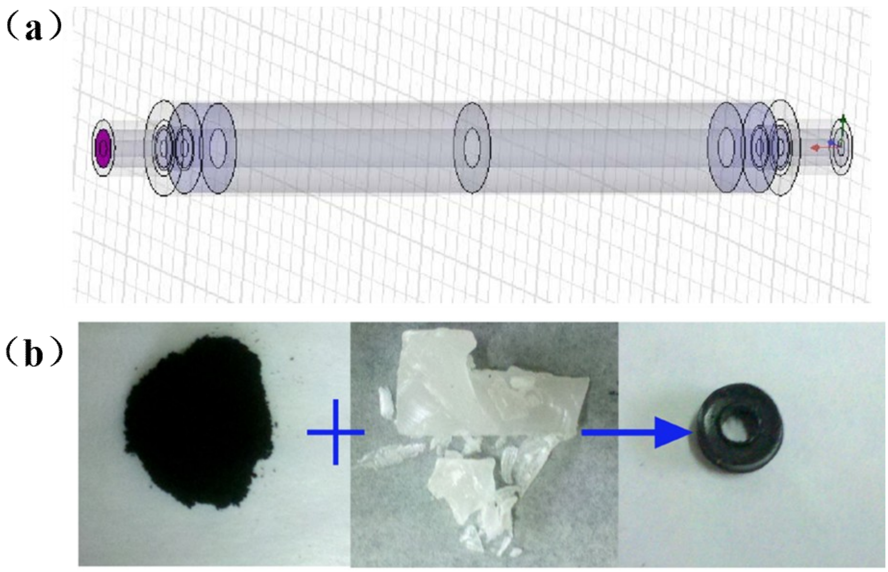

Figure S1. Schematic diagram of the fixture (a) and the test sample (b).

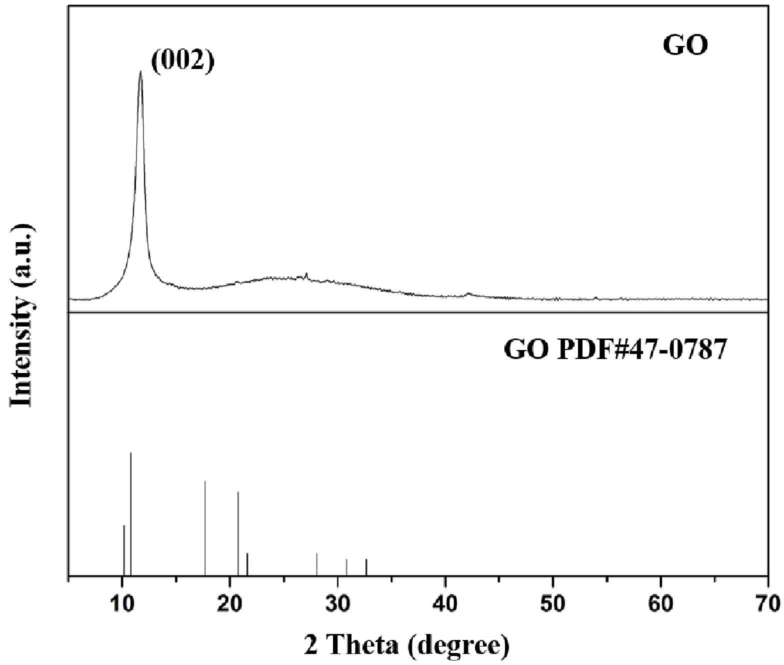

Figure S2. The XRD pattern of GO.

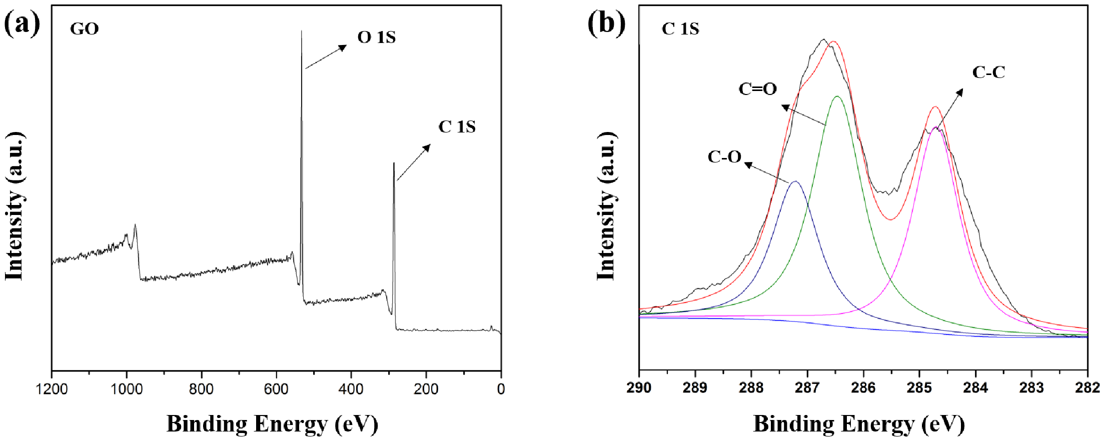

Figure S3. XPS spectra of GO (a); XPS core-level spectra of C 1s in GO (b).

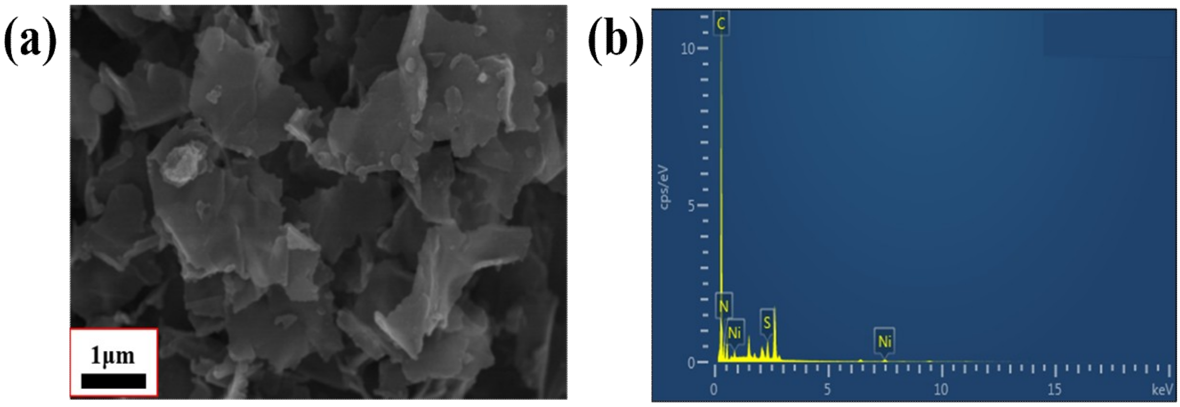

Figure S4. SEM image of NiS_2@rGO/PPy (a) and the corresponding EDS spectra (b).

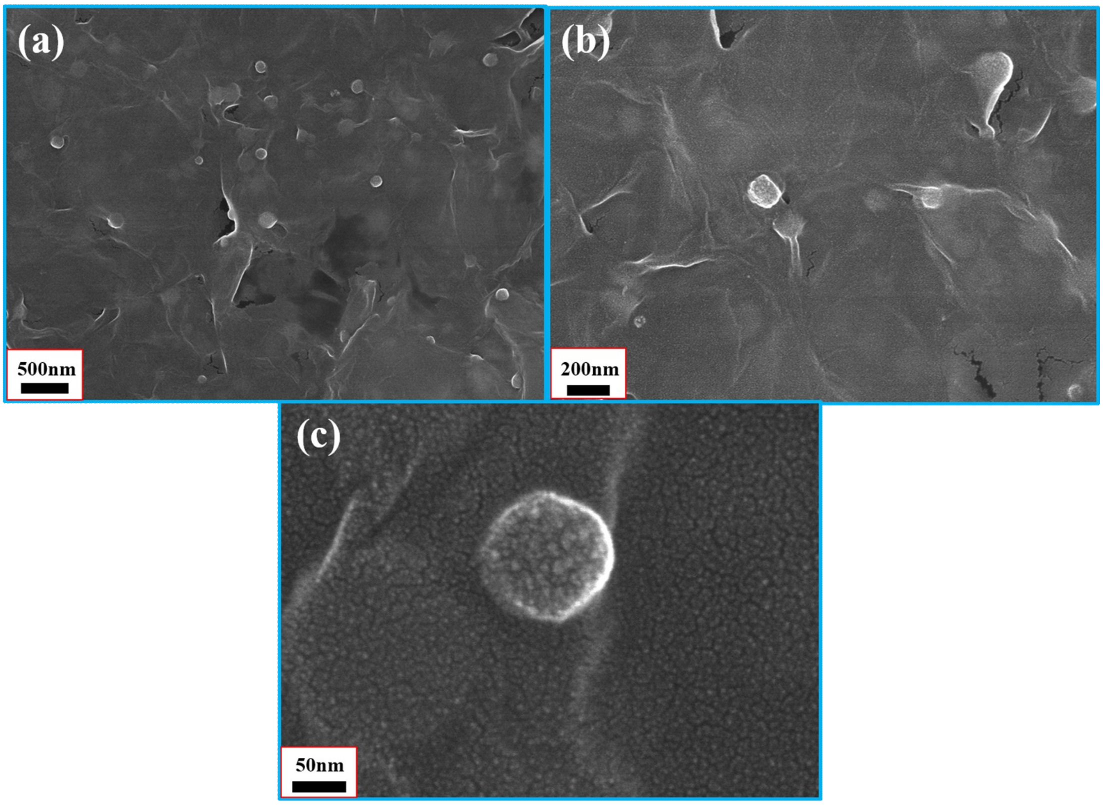

Figure S5. SEM images of NiS_2@rGO.
